# Ten simple rules for a successful international consortium in big data omics

**DOI:** 10.1371/journal.pcbi.1010546

**Published:** 2022-10-20

**Authors:** Miranda D. Stobbe, Abel Gonzalez-Perez, Nuria Lopez-Bigas, Ivo Glynne Gut

**Affiliations:** 1 CNAG-CRG, Centre for Genomic Regulation (CRG), Barcelona Institute of Science and Technology (BIST), Barcelona, Spain; 2 Institute for Research in Biomedicine (IRB Barcelona), The Barcelona Institute of Science and Technology, Barcelona, Spain; 3 Research Program on Biomedical Informatics, Universitat Pompeu Fabra, Barcelona, Spain; 4 Centro de Investigación Biomédica en Red de Cáncer (CIBERONC), Barcelona, Spain; 5 Institució Catalana de Recerca i Estudis Avançats (ICREA), Barcelona, Spain; 6 Universitat Pompeu Fabra (UPF), Barcelona, Spain

An African proverb says that “*If you want to go fast*, *go alone*, *if you want to go far*, *go together*.” There are many scientific challenges that exceed the possibilities of an individual laboratory, a single country or even a continent. Proving the existence of the Higgs boson particle would not have been possible without the huge international effort of the Large Hadron Collider (LHC) project [1]. This project brought together over 10,000 scientists from more than 100 countries around the world. Biology, however, in contrast to physics, has for a long time largely been a discipline of individual achievements. The Human Genome Project (HGP) [2] marked a key departure from an individualistic to a more collaborative approach in the field of biology. The HGP involved 20 institutes from 6 countries. Since then further large consortium projects in biology have been initiated. Each consortium united under a common goal ranging from providing fundamental information about genomes, e.g., International HapMap project [3], to tackling genomics of disease, e.g., the International Cancer Genome Consortium (ICGC) [4]. The cost of sequencing DNA has decreased dramatically over the last decade and consequently the ambition of consortia to generate even larger datasets has increased. In addition, existing datasets are being combined in new studies to tackle more complex questions.

In these large, international consortia funders often support their scientists locally and for many of these projects, participation depends on the level of funding committed. If a consortium does not have central funding, this poses an additional challenge of ensuring researchers live up to their promises without a “carrot and stick” at hand. The project also needs to rely on the resources consortium members provide, such as computing power, storage of the data, etc. The most critical resource, however, remains time. Time participants dedicate to the project is not enforceable when there is no central funding. The consortium may also not be the main project of the participants and the time they dedicate to it may vary. Personal motivation and engagement for the topic of the consortium may be the only things that keep the consortium going.

In hindsight, vision is always 20/20, therefore we looked back at consortia related to analysing omics data that we have been part of to see what we can learn from them. One consortium in particular in which we gained a lot of experience with a large international effort in the field of big omics, without central funding, was the ICGC/TCGA PanCancer Analysis of Whole Genomes (PCAWG) project [5]. This has been one of the largest biological projects aimed at getting the most out of combining existing datasets, jointly analysing nearly 2,700 cancer genomes. More than 1,300 scientists were involved from 37 different countries (https://www.icgc-argo.org/news/8/icgc-tcga-pan-cancer). We have summarised our experience into 10 rules as a guideline for future consortia dealing with large dataset and analyses, particularly when operating without central funding. Although these 10 rules should be obvious and may seem rather straightforward, most, if not all, are nevertheless often broken, ignored, or forgotten.

## Rule 1: Set up a transparent and effective governance

For a consortium to be successful, it needs to have a transparent and effective governance. The 2 key components of this are a clear set of rules and a well-defined organisational structure. The importance of a transparent set of rules, adhered to by all consortium members, is frequently overlooked. The rules should detail the organisational structure, what is expected from each participant and the foreseen outcomes of the consortium (Rule 2), with special emphasis in the publication and communication policy (Rule 9). For example, there should be clear rules on authorship. How will the long list of authors be shown? Who will be listed as named authors in the main paper? What are the rules to include authors in the companion papers? Another important part of the set of rules should be how to deal with foreseeable conflicts of interest. All these rules should be agreed upon before the actual start of the project. In this way, people can decide whether they would be willing and able to adhere to the rules defined. In centrally funded projects, this often adopts the form of a consortium agreement as part of the grant application. Grant providers like the European Commission provide documents with guidance on this (https://ec.europa.eu/research/participants/data/ref/h2020/other/gm/h2020-guide-cons-a_en.pdf). We should not forget a consortium agreement is mandatory for good reason, and even without central funding, there still should be something similar to it. Importantly, there should be no further changes to the rules once the project has started, unless major unforeseen circumstances warrant this. In which case, the majority of the consortium should support the change and thereby avoiding that important decisions that affect all are being made by just a few people.

For the second component of the governance of the consortium, the organisational structure, the division of the consortium into smaller working groups, especially in big consortia, should form the foundation. On top of that structure, there should be a steering committee. The working groups serve to organise scientific discussion around specific topics, do the analyses, and write the publications. The main tasks of the steering committee should be to enforce the rules in the day-to-day functioning of the consortium, ensure that the work within the consortium contributes to the overall scientific objectives (Rule 2), and is in line with its quality standards. The committee must guarantee the functioning of working groups and coordination among them. This is essential for the flow of ideas between scientists within the consortium, enhancing creativity, which is key to further science. They should make sure that the consortium achieves its goals in a timely manner (Rule 8). The steering committee should also provide a unified representation of the consortium in front of stakeholders, in particular scientific journals and the media, and defend the interests of the entire consortium. If the steering committee members are also scientific players in the consortium, this poses potential conflicts of interest. Although this is not ideal, in practice this may need to be the case as otherwise committee members do not get much in return for their effort and it is hard to follow all the ins-and-out of a project from progress meetings. The rules agreed upon should detail a way to deal with this double role of the steering committee.

## Rule 2: Know where you are going by setting clearly defined goals

The goals of the consortium need to be clearly defined, as failure to do so will have a ripple effect throughout many facets of the project. Clear goals are needed to be able to define and agree on the right terminology (Rule 3), to have proper time management (Rule 8), to define a publication strategy (Rule 9), and to ensure others can build upon the results obtained (Rule 10). Aligning with the goals of the project already starts at the data acquisition stage. Despite that it may be tempting to cast a wide net and collect as much data as you can get your hands on, irrespective of relevance, available metadata and quality, there are risks involved in this “the more, the better” strategy. Some of the data collected may not be relevant to answer the questions at hand or insufficient in quantity to answer questions nobody initially thought of. For example, if the main objective is to study primary tumours, including a handful of metastatic tumours is unlikely to be of any use. If irrelevant data is included, the main signal of interest of the project could be distorted or the interpretation of the results complicated. It could also lead to wasting valuable time and resources on samples that cannot be used later on. For similar reasons, it is also important to specify from the beginning the minimum required quality of samples for inclusion in the study, and what metadata is essential, the absence of which would lead to the exclusion of samples later.

## Rule 3: Use the right tools and terminology to communicate

**Fig 1:**
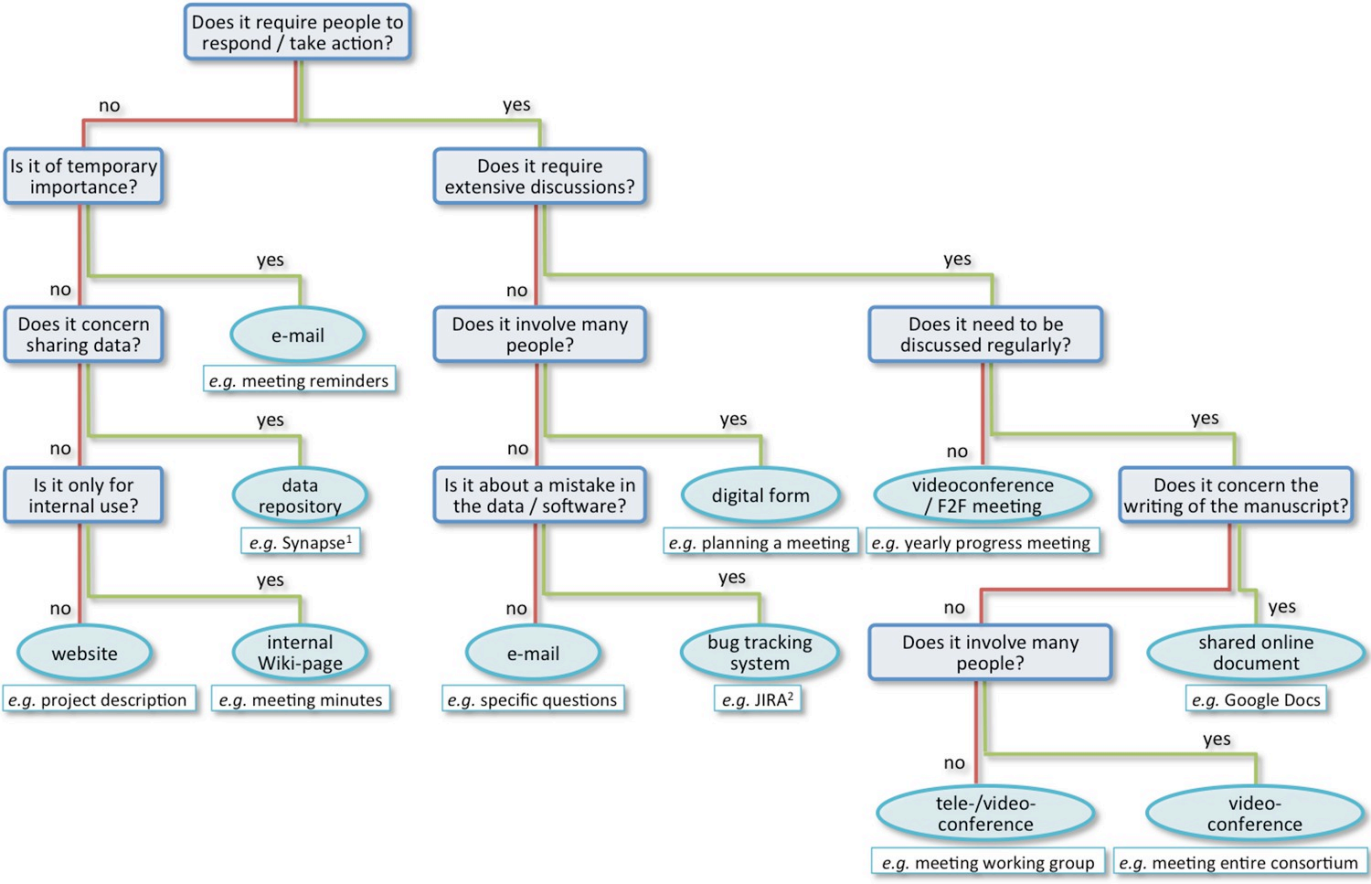
Communication tools and when to use them. Each communication tool has its own advantages and disadvantages. Sending an e-mail is not always the right solution and could result in an avalanche of messages. It is, however, also not necessary to have a tele-/videoconference for every little thing. This flowchart proposes a way to decide which tool is likely to be the most appropriate for the given purpose. Key is to keep in mind how much interaction is needed between the people involved and how large this group of people is. Generally, a videoconference is preferred over a teleconference, especially when the group of people involved is large. For some of the more specific points of communication like mistakes in data or software, there are specialised tools available. F2F: face-to-face. ^1^ Synapse: www.synapse.org. ^2^ JIRA: https://www.atlassian.com/software/jira.

**Fig 2:**
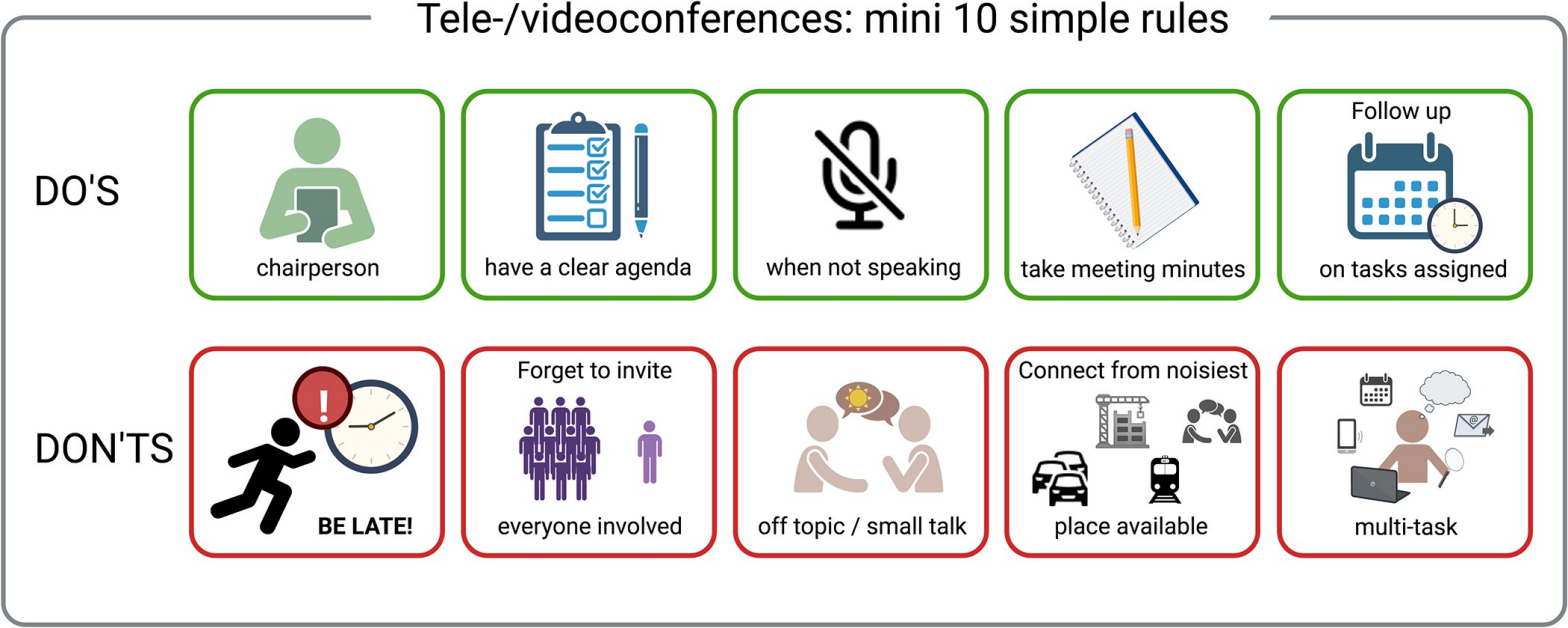
Mini 10 simple rules for tele-/videoconferences. If we could stick to these 10 simple rules during a tele-/videoconference, this type of meeting could be more efficient and less of a strain. This figure was partly created with BioRender.com except for the icons of the muted microphone, construction work, traffic, running late person, public transport, and e-mail. The icon of the muted microphone and traffic were downloaded from https://icons8.com/ and used in unmodified form.

Communication is essential to enable collaboration, exchange ideas, inspire others, and ensure everyone is on the same page. In projects involving hundreds of researchers from various backgrounds, efficient communication is challenging. It requires the use of the right communication tools and the development of a shared “language.” There are a multitude of tools available to facilitate collaborations among extended groups, some of which increased in popularity during the COVID pandemic. It is key, however, to choose the ones best suited for the type of information that needs to be conveyed (Fig 1). One also has to remain practical in choosing which resources to use to facilitate collaboration. These resources must be easily accessible, for example, avoiding the hassle of having to log onto several different systems and having to make yet another account. A few simple rules should be respected to guarantee the success of tele-/videoconferences (Fig 2). We would also suggest recording online conferences to share them across the consortium. Furthermore, a balance must be attained between the need for (scientific) discussions and the time this takes away from other tasks.

The challenge and importance of defining a shared “language,” i.e., identifiers and terminology, should not be underestimated. It is important to ensure everyone interprets terms in the same way and decisions in this regard should be made at the beginning of the project or when the term in question becomes relevant. Existing ontologies like those that can be found in the OBO Foundry (https://obofoundry.org/) could aid in this. Some leeway may need to be given for changes in terminology to accommodate new insights. However, one should keep in mind that once a certain identifier or term is introduced in a consortium of hundreds of people, it becomes next to impossible to change it. It acquires a life of its own, is used in programming code, manuscripts, figures, and so on. Trying to change things midway may result in multiple identifiers and terms being used next to each other, which in turn leads to confusion. At the publication stage, it is impossible to control that all manuscripts from the consortium adhere to the same terminology and identifier, if they have not been defined and agreed upon beforehand. Finally, as articles of large consortia are likely to turn into a reference by research projects to follow, ensuring the use of correct terminology and if necessary resolving any discordance in the field should be seen as the responsibility of the consortium and part of its legacy.

## Rule 4: Keep in mind you are collaborators, not competitors

In particular for very large consortia splitting into working groups makes communication easier within the groups and speeds up their work. However, there will always be a degree of overlap between working groups and results from one group can be important for other groups. Ultimately, all groups work on the same data. Consortium members should share all of their progress instead of keeping their cards close to their chest. It is important not to lose sight of the fact that within a consortium, you are all collaborators rather than competitors. Sharing the abstracts of proposed manuscripts with the entire consortium as early as possible is a good idea to ensure there are no duplicate efforts and potential collaborations could be discovered. One note of care: Although there is nothing wrong with getting inspired by the work of others, credit should be given where credit is due and mindfulness is important to not give the impression ideas were stolen.

## Rule 5: Build in checkpoints at every stage of the project

As the famous principle in computer science goes, “Garbage in, Garbage out.” This holds true for every stage of a project, from the primary data down to, for example, mutation calls. Examples of what to check at the various stages are listed in Table 1. It is key to detect any issues with the input at every stage in terms of quality and potential errors as early as possible. It is also important to be aware of missing data and record why it is missing. Not detecting any structural mutations in a tumour sample is not the same as a pipeline unable to call structural mutations for this specific sample due to technical issues. In case the missing values can be imputed and there is a desire to do so, you need to know whether or not they are missing at random [6]. By detecting issues early, you can prevent resources from being wasted on analysing samples that do not meet quality standards and prevent errors from propagating to downstream analyses. Having to rerun analyses is not only demotivating, it is also time and resource consuming. Moreover, rerunning may be a luxury a consortium cannot afford, forcing it to adopt an imperfect solution instead. Working with ever-changing input data is frustrating for researchers involved in downstream analyses. In addition, having multiple versions of a certain (derived) dataset floating around may lead to different groups using disparate versions. Definite data freezes are very important.

**Table 1:** Examples of sanity checks in a human cancer genomics project.

Stage	Examples of sanity checks
Data acquisition — omics data	◾ sample is relevant to the goals of the project ◾ sufficient quality ◾ tumour and normal sample are from the same donor ◾ donor is not submitted multiple times with different IDs ◾ multiple aliquots from the same sample are indicated as such
Data acquisition — clinical data	◾ sufficient clinical data is available for the goals of project ◾ sex of donor coincides with genome ◾ for sex-specific tumour types: corresponds with sex of donor ◾ all values are in the correct range, e.g., survival time is >0 days ◾ same unit is used across samples, e.g., survival time is in days for all ◾ one word, same spelling, same capitalization for the same term, e.g., breast and not also mamma/mammae/Breast/etc.
Primary data generation (e.g., mutation calls)	◾ VCF files are correctly formatted ◾ sufficient quality after read alignment [7] ◾ PCR artefacts [8,9] ◾ FFPE^1^ artefacts [10] ◾ oxidative artefacts [11] ◾ high % of paired reads mapping to different chromosomes^2^ ◾ purity of the tumour sample ◾ high % of tumour reads in normal sample ◾ contamination with foreign DNA (e.g., other individuals, mouse) ◾ contamination with RNA ◾ make sure there is no over- or underfiltering of mutation calls ◾ missing data
Downstream analysis — 1 … Downstream analysis — n	◾ outliers ◾ all downstream analyses use the same version of the data ◾ tools developed work in various compute environments ◾ conclusions are not solely based on samples with lower quality ◾ sensitivity of results to (slight) changes in parameters
Before submitting manuscripts	◾ contradictory results across analyses ◾ across all manuscripts same basic statics, e.g., number of samples

^1^ Formalin-fixed, paraffin-embedded.

^2^ Although there are biological reasons for this to happen, such as chromothripsis, if this percentage goes above 3%, it is more likely a technical artefact [7].

Samples that in hindsight should have been excluded, must not be kept for the sole reason that resources have already been spent on them. It is important to know when to cut your losses. Low-quality samples will introduce noise, while adding very little—if any—useful information to the analysis. In a project where data is collected from different sources that use different protocols, quality is likely to vary, which is important to be aware of and to quantify [7]. This allows researchers to potentially correct for batch effects and be confident that their conclusions are not just reflecting differences in quality between samples or are based on lower quality samples.

Finally, before data or a tool is shared with the entire consortium, the responsible working group has to ensure there are no mistakes in it. However, it is not easy to spot your own mistakes. Using the entire consortium as a testing ground may increase the chances of mistakes being identified, but it is also a costly strategy. An initial check by a selected test team, not involved in the analysis in question, can help prevent many errors from burdening the project. This would be similar to the dedicated test teams in commercial companies.

## Rule 6: Learn how to walk before you run

In a “wet-lab” setting, you would never use all your starting material at once without going through several testing phases on small amounts of material to ensure every step of the process works. In bioinformatics analyses, however, there is the tendency to run pipelines immediately on the entire dataset. For the primary analyses, like alignment and mutation calling, the goal is to allow analyses downstream to start as soon as possible. As no physical material is being wasted, it seems this can be done with impunity, but in reality it might result in wasting (computational) resources. Slow and steady is more likely to win the race. The effort to apply someone else’s software tool, even a published one, should not be underestimated. The developer of a tool is often the most adept user. Software is often developed and tested in a specific computer environment. Moreover, there are multiple settings in a tool that have to be tuned for the dataset at hand.

Running the workflow initially on a subset of the data also makes it easier to detect if the chosen terminology is adequate (Rule 3), to detect inconsistencies, and to ensure no errors have been made (Rule 5). This is especially important for the primary analyses as they form the foundation for all subsequent analyses and having to redo this part will have far-reaching consequences. The initial test set should be as representative as possible of the entire set of samples and once it runs smoothly without apparent errors, a first release can be shared with the consortium for downstream analyses. From here on, one can start to run the workflow on an increasingly larger dataset with intermediate, incremental releases to the consortium. At this point the workflows, tools, and terminology used should be frozen, as continuous changes to these things make it impossible to continue with the next step. Note that some errors may only come to light with particular samples, which is why a test team is essential (Rule 5). If multiple tools are applied to the same data and subsequently a consensus is built, then it is important to run the samples in the same order through the pipelines.

## Rule 7: Keep track of code and data provenance

For the purpose of data provenance and as part of adhering to the Findable, Accessible, Interoperable and Reusable (FAIR) principles [12], what should be stored ranges from which institute sequenced a sample to the “in silico” processing steps. This is even more important when the data comes from many different sources each with their own system in place. This may lead to, among other things, a multitude of identifiers for the same sample. The facility that collects the sample attaches an identifier, so does the sequencing facility and if submitted to a public repository another identifier is linked to it to make the sample unique in a cross-project platform. Another one might even be added to give the sample a universally unique identifier (UUID) [13], which would be the preferred one to use, although due to its length it is impractical for use in article figures. Purely for the use in figures, the identifier assigned by the (public) repository could serve as an alternative to the lengthy UUID. Each identifier linked to a sample at any stage should be recorded and shown in the repository for traceability. In the case of multi-omic data, it is also essential to ensure the identifiers accurately reflect whether the different types of omics were derived from the exact same sample or only the same donor.

Recording data provenance makes it possible to trace back the origin of anomalies and quality issues detected further down the process. For example, the use of PCR amplification in the sample preparation increases specific artefacts [8,9]. Having the use of PCR amplification recorded is better than having to discover this post hoc. Knowing which analyses were done, the settings used and filters applied are all important as it allows everyone to understand how results need to be interpreted and the potential limitations of the data. It also makes it easier for others to reproduce results and build upon them (Rule 10). There are various tools available to keep track of software that is being developed and facilitate a clear description of the workflow. Examples are Git for version control (https://git-scm.com/) and the virtual environment management system Conda (https://docs.conda.io/en/latest/). Workflow managers like Jip (https://pyjip.readthedocs.io/en/latest/index.html), Nextflow [14], and Snakemake [15] can provide a way to describe the steps and share with others. Also advisable is sharing files containing data and intermediate results across working groups, using platforms such as Synapse [16], capable of tracking file provenance and versioning. In summary, wise use of existing tools may go a long way to guarantee data provenance and software availability.

## Rule 8: Keep up the pace and finish within a reasonable timeframe

Time management is especially complicated in projects without central funding, as there is no overall accountability and no mandatory reporting in place to put pressure on people to deliver their contribution to the project. Also, the availability of resources depends entirely on what participating groups can offer and it is difficult to estimate the time people will be able to invest. The time needed for the “blue collar” part of a project is often underestimated, for example, the logistics behind sharing omics data and the potentially associated legal issues. For large datasets, sufficient bandwidth to transfer the large volumes of data needs to be taken into account. As advocated by Stein and colleagues [17], data should be available in a more accessible place like a cloud. Taking the computation to the data avoids transferring data. However, this might come at the cost of having to invest time to make a tool work in a different compute environment than in which it was developed. Although this process is often more time-consuming than expected (Rule 6), it is worthwhile for large datasets, especially given the added legal issues of moving data out of the place where it was generated. Producing and distributing software that can seamlessly be run across platforms is also a very important responsibility of large consortia (Rule 10).

There may not be a set end date of the entire project aside from the data embargo that may need to be lifted at some point. This can be problematic, especially for PhD students and postdoctoral fellows, as they often have short-term contracts and are under time constraints to publish their work. Delays in publishing the main consortium article might hold back companion articles. An effective mitigation strategy is to go for a more flexible publication policy, where you do not wait for the main consortium paper to be accepted first (Rule 9). It is also important to consider that the novelty of the project’s findings decreases with time. Delays may thus diminish the impact of the results.

Even without externally imposed deliverables by funding agencies, deadlines have to be set and met. It is important to establish a realistic timeline and abandon elements that hold back the group if they are not essential for the project. Laying down overly aggressive timelines and then letting them slip, reduces the enthusiasm of the group and leads to frustration. Consortium members must refrain from overpromising results, and must spend the time on the project as promised, and complete the tasks assigned to them (Rule 1). Even if there is no funding party imposing penalties for not sticking to what was written in the original proposals, there needs to be discipline among the consortium members.

## Rule 9: Define an effective and fair publication strategy

The basic principles behind an effective and fair publication strategy are simple: ensure the science is communicated clearly and that credit is given where credit is due. Failure to meet the expectations of consortium members in terms of publications is demotivating and leads to frustration. Proper credit should be given not only to those with tenure, but also especially to those without it, i.e., PhD students and postdocs. The mere existence of the aphorism “publish or perish” is a testament to the importance of publications. The number of articles, the journals where they are published, the length of the author list, and the position each person has in it are all important for the evaluation of scientific performance. While this system may be flawed in many aspects, it is the one in place to communicate scientific results. One could rightly argue that large consortia could have the power to bring around important changes in the system, including setting the requirements that the main consortium article should satisfy.

The shape and content of this main article may be a particular point of contention between a consortium and a journal. It is in the interest of journals to propose to put all or most of the results in a single publication that attracts many readers and citations, thus increasing their impact factor. However, cannibalizing companion articles is detrimental to the principle of clarity. It is hard for the authors to explain their stories in a clear way in a long article with many and diverse results intermingled. These dense and long articles are also very difficult to digest by the reader. A lot of relevant information is relegated to the supplementary section, which steals the thunder of companion articles. The main consortium article also enters into conflict with the principle of giving proper credit to authors, as a long list of authors under a consortium name, with few highlighted as named authors or corresponding authors, does little justice to the time and effort put in by PhD students and postdocs. The particle physics community lists authors in alphabetical order and at least in this way provides equity.

One solution to this problem is that the main consortium article provides a global perspective on the project, the datasets generated, the technical challenges faced and their solutions, while the scientific discoveries are left to companion articles, which the main article cites. The companion articles with fewer authors and a focused story with a clear message are likely to be of more value to the scientific community (writers and readers). Such a strategy would also free companion articles for publication as they become ready, rather than forcing them to wait for the publication of the main consortium article. The main article would still be highly cited, as researchers would refer to it as the source of the data and when citing the main achievements of the consortium. An early, skeleton version of the main article could serve as a point of reference within the consortium to ensure all basic information, like the number of samples, and terminology is the same across all companion papers.

There is little use to start negotiations with one specific journal before there is a clear idea of the outcome of the project. This just increases the chance of creating unrealistic expectations for both parties. Instead throughout the project it should be evaluated what the best strategy will be with the agreement of consortium members. Too close collaboration between a journal and a consortium jeopardizes the fairness of the peer review process as the willingness of the journal to accept a manuscript will be higher than normal.

Another recommendation is to publish the results as preprints as soon as they are ready to be shared with the scientific community, which helps speed up the scientific process. As a preprint can already be cited, even at this stage it is important to give credit to all involved and to include the contributions of each person, which is in any case a requirement of many journals. This also prevents some people from being authors on all articles coming out of the consortium, while others appear on less than they should. This does require everyone to be realistic about their contribution.

Finally, a great resource to provide valuable feedback and further improve the quality of a manuscript before submitting it, are those consortium members that were not involved in the particular manuscript. A system should be in place to ensure that the work that comes with this is equally distributed across the consortium members and that for each manuscript the members with the right expertise are asked to provide feedback. What should be avoided, however, is that, for example, the steering committee takes the role of reviewer, preventing manuscripts from being submitted and thereby interfering with the normal peer-review process. It also puts all the power regarding the publication strategy in the hands of just a few people (Rule 1) and can cause delays, compromising the timely publication of results (Rule 8). Moreover, if the members of the steering committee are also involved in multiple subprojects, this is a source of a potential conflict of interests or at the least appearance of it. In this regard, the journals are still the ones responsible for selecting unbiased and sufficiently experienced reviewers, and although the pool of possible reviewers may be depleted in the case of large consortia, there will always be qualified external reviewers able to do the job.

## Rule 10: Be the giant on whose shoulders others can stand

It is key that future projects can build on the results produced by the consortium. Even before starting your project, you should think about what the next consortium might need to be able to continue where you left off (Rule 2). For example, if the project involves sequencing data, it is worth doing sequence alignment to the most recent version of the genome reference even if it at the moment is not the one most commonly used. If this is not done, it could mean the data becomes obsolete faster. Or if the next project wants to use it, a lot of work might have to be put in to bring the data up to date. Alternatively, a new project may choose to use the data as is to avoid this, which basically sets back the field as it is not using the improved version of the reference genome.

Sharing the results in the form of articles is only one way for a consortium to leave a legacy. As detailed in the 10 simple rules of Boland and colleagues [18], it is also essential to share data and tools such that others can build on it. A common phenomenon in bioinformatics is that tools mainly seem to work for the people who developed them. Others trying to use them often encounter difficulties and need to invest significant time to get them to run. This is why it is so important to document all the tools used, make them available in a format that everyone can use and when applied to the same data will get the same answers. To ensure that the workflow is portable between different operating systems, container technologies such as Docker (https://www.docker.com/) or Apptainer (https://apptainer.org/) may be used. Code can be documented and shared using among others, Jupyter notebooks (https://jupyter.org/index.html), Rmarkdown [19], and open source repositories like Code Ocean [20] and Zenodo (https://zenodo.org/). Optimising the tools in terms of speed and memory usage is also worth considering given that datasets will continue to grow and thereby the return on the investment increases. It will be a valuable legacy for future endeavours.

Aside from the software, it is also important to share the data along with the associated metadata and data provenance (Rule 7). This allows the next project to build on top of it and take the field to the next level. All of this taken together can in short be summarised as that you need to make sure you adhere to the FAIR principles [12].

## Concluding remarks

Key for a successful consortium is to ensure you indeed go farther and not just slower. There is a real risk that in failing to keep up the pace, a large-scale consortium can hold back the field rather than stimulate progress. A consortium should be a platform for the development and promotion of new ideas. The data and findings should also not be held hostage for the glory of a few. Making an effort to stay friends is of great importance as well. Building consortia to tackle big research questions is key to advance science. We hope that these 10 simple rules may help future consortia to indeed go farther and build networks that are enriching, particularly for young scientists.

## References

[1] Aad G, Abajyan T, Abbott B, Abdallah J, Abdel Khalek S, Abdelalim AA, et al. Observation of a new particle in the search for the Standard Model Higgs boson with the ATLAS detector at the LHC. Physics Letters B. 2012;716(1):1–29. doi: 10.1016/j.physletb.2012.08.020

[2] Venter JC, Adams MD, Myers EW, Li PW, Mural RJ, Sutton GG, et al. The Sequence of the Human Genome. Science. 2001;291(5507):1304–1351. doi: 10.1126/science.1058040 11181995

[3] Gibbs RA, Belmont JW, Hardenbol P, Willis TD, Yu F, Yang H, et al. The International HapMap Project. Nature. 2003;426(6968):789–796. doi: 10.1038/nature02168 14685227

[4] Hudson TJ, Anderson W, Aretz A, Barker AD, Bell C, Bernabé RR, et al. International network of cancer genome projects. Nature. 2010;464(7291):993–998. doi: 10.1038/nature08987 20393554 PMC2902243

[5] Consortium ITP-CAoWG. Pan-cancer analysis of whole genomes. Nature. 2020;578(7793):82–93. doi: 10.1038/s41586-020-1969-6 32025007 PMC7025898

[6] Song M, Greenbaum J, Luttrell J, Zhou W, Wu C, Shen H, et al. A Review of Integrative Imputation for Multi-Omics Datasets. Front Genet. 2020;11. doi: 10.3389/fgene.2020.570255 33193667 PMC7594632

[7] Whalley JP, Buchhalter I, Rheinbay E, Raine KM, Stobbe MD, Kleinheinz K, et al. Framework for quality assessment of whole genome cancer sequences. Nat Commun. 2020;11(1):5040. doi: 10.1038/s41467-020-18688-y 33028839 PMC7541455

[8] Gao R, Zhao AH, Du Y, Ho WT, Fu X, Zhao ZJ. PCR artifacts can explain the reported biallelic JAK2 mutations. Blood Cancer J. 2012;2(2):e56–e. doi: 10.1038/bcj.2012.2 22829246 PMC3288281

[9] Alioto TS, Buchhalter I, Derdak S, Hutter B, Eldridge MD, Hovig E, et al. A comprehensive assessment of somatic mutation detection in cancer using whole-genome sequencing. Nat Commun. 2015;6(1):10001. doi: 10.1038/ncomms10001 26647970 PMC4682041

[10] Do H, Dobrovic A. Sequence artifacts in DNA from formalin-fixed tissues: causes and strategies for minimization. Clin Chem. 2015;61(1):64–71. doi: 10.1373/clinchem.2014.223040 25421801

[11] Costello M, Pugh TJ, Fennell TJ, Stewart C, Lichtenstein L, Meldrim JC, et al. Discovery and characterization of artifactual mutations in deep coverage targeted capture sequencing data due to oxidative DNA damage during sample preparation. Nucleic Acids Res. 2013;41(6):e67–e. doi: 10.1093/nar/gks1443 23303777 PMC3616734

[12] Wilkinson MD, Dumontier M, Aalbersberg IJ, Appleton G, Axton M, Baak A, et al. The FAIR Guiding Principles for scientific data management and stewardship. Scientific Data. 2016;3(1):160018. doi: 10.1038/sdata.2016.18 26978244 PMC4792175

[13] Leach P, Mealling M, Salz R. A Universally Unique IDentifier (UUID) URN Namespace. RFC 4122. 2005. doi: 10.17487/RFC4122

[14] Di Tommaso P, Chatzou M, Floden EW, Barja PP, Palumbo E, Notredame C. Nextflow enables reproducible computational workflows. Nat Biotechnol. 2017;35(4):316–319. doi: 10.1038/nbt.3820 28398311

[15] Mölder F, Jablonski K, Letcher B, Hall M, Tomkins-Tinch C, Sochat V, et al. Sustainable data analysis with Snakemake. F1000Research. 2021;10(33). doi: 10.12688/f1000research.29032.2 34035898 PMC8114187

[16] Omberg L, Ellrott K, Yuan Y, Kandoth C, Wong C, Kellen MR, et al. Enabling transparent and collaborative computational analysis of 12 tumor types within The Cancer Genome Atlas. Nat Genet. 2013;45(10):1121–1126. doi: 10.1038/ng.2761 24071850 PMC3950337

[17] Stein LD, Knoppers BM, Campbell P, Getz G, Korbel JO. Data analysis: Create a cloud commons. Nature. 2015;523(7559):149–151. doi: 10.1038/523149a 26156357

[18] Boland MR, Karczewski KJ, Tatonetti NP. Ten Simple Rules to Enable Multi-site Collaborations through Data Sharing. PLoS Comput Biol. 2017;13(1):e1005278. doi: 10.1371/journal.pcbi.1005278 28103227 PMC5245793

[19] Xie Y, Allaire JJ, Grolemund G. R markdown: The definitive guide. Chapman and Hall/CRC; 2018. doi: 10.1201/9781138359444

[20] Clyburne-Sherin A, Fei X, Green SA. Computational Reproducibility via Containers in Psychology. Metabolism. 2019;3. doi: 10.15626/MP.2018.892

